# Using marketing theory to inform strategies for recruitment: a recruitment optimisation model and the txt2stop experience

**DOI:** 10.1186/1745-6215-15-182

**Published:** 2014-05-22

**Authors:** Leandro Galli, Rosemary Knight, Steven Robertson, Elizabeth Hoile, Olubukola Oladapo, David Francis, Caroline Free

**Affiliations:** 1 London School of Hygiene and Tropical Medicine, Coventry CV4 7AL, UK; 2 Department of Medical Statistics, Faculty of Epidemiology and Population Health, London School of Hygiene and Tropical Medicine, London WC1E 7HT, UK; 3 Centre for Research and Innovation Management, University of Brighton, Brighton BN2 0JG, UK; 4 Department of Population Health, Clinical Trials Unit, Faculty of Epidemiology and Population Health, London School of Hygiene and Tropical Medicine, London WC1E 7HT, UK

**Keywords:** Marketing mix, Marketing model, Recruitment performance, Social marketing, Turnaround, Txt2stop

## Abstract

**Background:**

Recruitment is a major challenge for many trials; just over half reach their targets and almost a third resort to grant extensions. The economic and societal implications of this shortcoming are significant. Yet, we have a limited understanding of the processes that increase the probability that recruitment targets will be achieved. Accordingly, there is an urgent need to bring analytical rigour to the task of improving recruitment, thereby increasing the likelihood that trials reach their recruitment targets. This paper presents a conceptual framework that can be used to improve recruitment to clinical trials.

**Methods:**

Using a case-study approach, we reviewed the range of initiatives that had been undertaken to improve recruitment in the txt2stop trial using qualitative (semi-structured interviews with the principal investigator) and quantitative (recruitment) data analysis. Later, the txt2stop recruitment practices were compared to a previous model of marketing a trial and to key constructs in social marketing theory.

**Results:**

Post hoc, we developed a recruitment optimisation model to serve as a conceptual framework to improve recruitment to clinical trials. A core premise of the model is that improving recruitment needs to be an iterative, learning process. The model describes three essential activities: i) recruitment phase monitoring, ii) marketing research, and iii) the evaluation of current performance. We describe the initiatives undertaken by the txt2stop trial and the results achieved, as an example of the use of the model.

**Conclusions:**

Further research should explore the impact of adopting the recruitment optimisation model when applied to other trials.

## 

“Marketing is a learning game. You make a decision. You watch the results. You learn from the results. Then you make better decisions”.

Philip Kotler

## Background

Randomised controlled trials are the gold standard for assessing health care interventions as they provide the most powerful research method for minimising bias [1]. Under-recruitment reduces trial power and results in imprecise trial effect estimates [2]. This can lead to a failure in detecting modest but significant clinical benefits. Recruitment remains a major challenge for many trials [3] as highlighted by McDonald et al. [1], who studied recruitment to multicentre trials between 1994 and 2002. They reported that only 31% achieved their original recruitment targets and 53% requested grant extensions. More recently Sully et al. [4] found that recruitment has improved, but about 45% of trials still struggle to recruit their sample size and approximately a third are forced to resort to grant extensions. It remains unclear why some trials succeed while others fail as the inter-relationship between trial characteristics and successful recruitment is complex [5]. The UK’s Medical Research Council has recognised that failure to recruit can be attributed to an inability to resolve practical problems rather than to scientific or trial design issues [6]. In order to improve recruitment rates there have been calls to apply greater analytical rigour and more insightful management practices [7] including the suggestion that the performance of clinical trials could be improved by looking beyond the world of clinical practice, specifically, by using marketing principles [7-9].

### Marketing and trials

**Figure 1:**
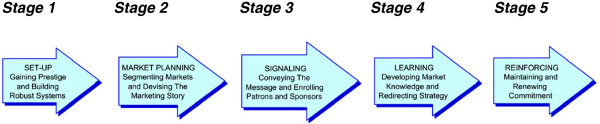
Five stages in marketing a trial.

Barriers to participation in trials begin with the kind of commitment that is required. Participants must commit to following a set of procedures, often involving additional effort and expense but without the assurance of receiving any direct benefits. Further, communication can be a barrier as participants can have difficulties comprehending the meaning of terms such as randomisation and equipoise [10]; such barriers can make trial participation seem unattractive. Marketing, as a discipline, focuses on meeting customers’ needs through the deep understanding of the factors that influence purchasing or sign-up decisions (in this case trial participation). Social marketing is the application of marketing principles for social benefit [11] and has been used in public health for more than 30 years, but mainly to improve promotional or communications activities [12,13]. The potential for marketing constructs to be used to improve the management of clinical trials remains largely unexplored, although Francis et al. [9] have suggested the use of marketing models and techniques beneficial for trialists and which can be understood as a five-stage process (Figure 1).

For those managing a trial, it can be valuable to have a mental model of the required marketing stages. Yet, while it is useful to know the necessary steps to marketing a trial, this is different from understanding the range of activities needed to optimise recruitment. The task of improving recruitment needs to be an iterative learning process [14-17] and no guiding theoretical framework is available to assist this process of targeted learning.

### Background to txt2stop

Our case study was txt2stop, a single blinded, randomised, control behaviour change trial that sought to establish the effects of supportive text messages on the rate of smoking cessation [18]. The trial was scheduled to recruit 5,800 participants over a 2-year period. Eight months into the trial, the number of participants that were randomised was 22% below target. At such a rate, recruitment would have been completed two years behind schedule and the costs would have exceeded the budget. However, the trial management team were able to identify and address the problems affecting recruitment and complete the trial four months early. This turnaround provides a valuable case that informed the development of the recruitment optimisation model.

### Study purpose

We aimed to generate a new, or to refine an existing trials-orientated marketing framework, based on the analysis of the txt2stop case and relevant literature from social marketing and clinical trials, that can be used by other trialists to improve recruitment.

## Methods

### Research methodology

The Francis et al. five-stage model [9] (Figure 1 and Table 1) was the conceptual framework of reference. Txt2stop recruitment processes were compared against the model and these insights were supplemented with a literature study of social marketing theory by LG. Studies were sought using academic databases. Emerald, Web of Science (Web of knowledge), Science Direct, PsychINFO, PubMed, BioMedCentral, Cochrane Library, and the internet (Google Scholar), were searched using the keywords: “social marketing”, “marketing clinical trials”, and “trial recruitment” for the time period 1960 to 2011. LG conducted a semi-structured interview with the principal investigator (CF) obtaining detailed information on the measures adopted to improve recruitment. A semi-structured approach to interviewing allowed new concepts to emerge from the interview. Notes were taken during the interview. Additionally, trial documents (promotional and communication material) and recruitment process data were reviewed.

**Table 1:** Activities within the five stages in marketing a trial

**Stage**	**Marketing purposes**
Set-up	1. To gain the buy-in of the necessary authorities and stakeholders.
	2. To gain the buy-in of opinion leaders whose explicit approval provides legitimacy and prestige for the trial.
	3. To construct a marketing function within the trial and devise robust systems for ensuring that the marketing (and later sales) activities are undertaken efficiently, effectively, and in accordance with the values and goals of the trial.
Market planning	1. To identify and describe the distinctive features of the ‘segments’ of the ‘market’ to be targeted.
	2. To discover what people in each of the selected market segments value (i.e., what would encourage them to ‘sign-up’).
	3. To develop a ‘value proposition’ (or more than one if required) that can be tested with each of the targeted segments.
	4. To enrol the whole trial organisation in working within the trial’s ‘marketing brief’.
Signalling	1. To convey, fully and persuasively, the ‘value proposition’ to sufficient numbers of people in the target market.
	2. To convey, fully and persuasively, the ‘value proposition’ to intermediaries (e.g., doctors or nurses), influencing bodies (e.g., ethics committees), and other agents that can either help or hinder the conduct of the trial.
Learning	1. To learn, through doing, about ‘the market’.
	2. To utilise ongoing learning to develop more effective policies and practices.
	3. To evaluate and redirect the strategy of a trial as learning is acquired.
Reinforcing	1. To maintain momentum by renewing or upgrading ‘the offer’ made to participants.
	2. To sustain commitment of interested parties and other agencies whose support will be needed.

## Results

### The model – “Old versus New” and its key marketing concepts

**Figure 2:**
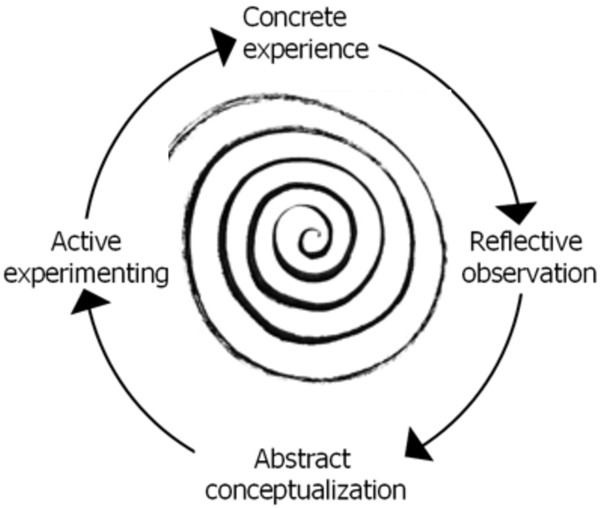
Authors’ representation of the Kolb learning spiral.

**Figure 3:**
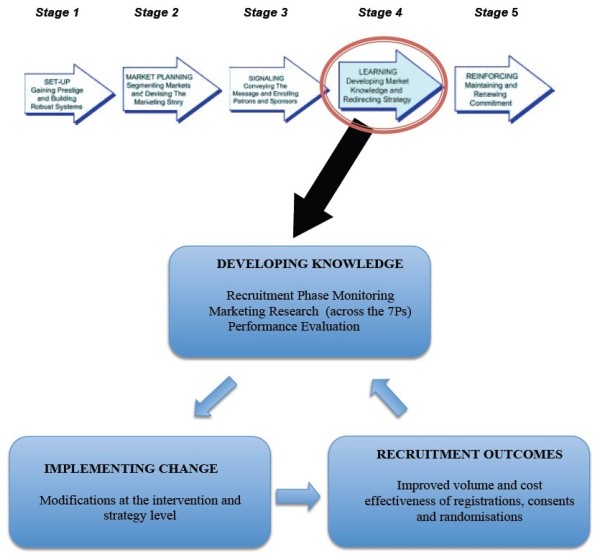
The previous five-stage model and the recruitment optimisation model.

The recruitment optimisation model is more dynamic than Francis et al.’s approach as it uses a Kolb-style framework in which multiple learning events take place as emergent and on-going processes that are grounded in experience [19]. The importance of dynamic learning capability was identified from an analysis of data, especially that from the semi-structured interview with the principal investigator (CF) whose description of the measures adopted by txt2stop was consistent with a Kolbian learning cycle (Figure 2). The trial’s current recruitment performance (successes and difficulties) was assessed through data collection practices (providing concrete experience). Performance data was compared against the trial’s goals and benchmarks (resulting in reflective observation). Social marketing constructs and insights from the clinical trial literature aided the process of sense-making (abstract conceptualisation). New or improved interventions and strategies were developed from a deeper understanding, and then tested and implemented (active experimentation). The outcomes from these initiatives provided further data that started a new learning cycle. Hence, as can be seen in Figure 3, the recruitment optimisation model is spiral and not linear, where feedback loops drive improvements in strategies and practices. The recruitment optimisation model focuses on the importance of managing this learning process by adopting appropriate behaviours for each of Kolb’s stages of the learning cycle.

The Francis model [9] (Figure 1) emphasised the importance of developing deep market knowledge and redirecting strategy in the light of experience, but did not describe the range of activities that would ensure adequate knowledge is developed. Drawing on key concepts from the social marketing literature [12,13,20-23] as well as the txt2stop experience, we found that learning involves four main tasks:

1. Recruitment phase monitoring

2. Marketing research

3. Performance evaluation

4. Change implementation

The four tasks are interdependent and when combined they enable trialists to better evaluate recruitment performance, to identify and understand recruitment problems and opportunities for improvement, and to successfully implement change initiatives that improve recruitment rates. Each task is described in turn, with examples from txt2stop to provide practical illustrations that explain the concepts.

### TASK 1 – recruitment phase monitoring

Trials have several recruitment phases that participants must progress through to be randomised. The concept is mentioned by Dyas et al. [15] who argue that recruitment should be considered in two distinct phases: i) getting potential participants to contact the trial and ii) converting contacts into consents. Within each phase there can be a number of steps. For instance, in the case of txt2stop, phase two had two steps.

•Step One: determining via phone, the eligibility of participants who had previously expressed an interest in the trial.

•Step Two: obtaining eligible participants’ consent via SMS.

It was found that the tracking of participants’ progress within and between phases was essential to allow any bottlenecks in recruitment to be identified.

### TASK 2 – marketing research

Marketing research assists trialists to identify market segments (categories of prospective participants), resulting in a greater understanding of their distinctive characteristics. This enables trialists to devise segment-specific strategies that are focused on the specific needs and wants of each category. Segments can be defined as homogenous sub-sets of a market, comprised of people who share similar wants and needs [13].

Marketing research provides a disciplined tool-kit for devising tailored solutions, by ascertaining if the ways in which the trial is being sold satisfies the specific needs and wants of each target market segment. This analysis can be complex as a trial’s target market may consist of several segments, to whom trial participation needs to be sold. A trial may need to be sold to trial participants themselves, as with txt2stop, or in other cases, to their legal representatives [24]. Further, market analysis is used to provide insights into other stakeholders whose involvement can be vital to a trial’s effectiveness, including its promotion. Stakeholders include trial facilitators, intermediaries/collaborators, gatekeepers, etc. This was true for txt2stop whose stakeholders (GPs, pharmacists, and smoking cessation services) were involved in the trial’s promotional efforts [18].

### The 7 Ps of the marketing-mix for trial participation

The marketing mix is the framework used for gaining a detailed understanding of the target market’s attitudes towards the trial. It is the “*the group of variables that a marketer can alter to successfully sell a product (good or service)*” ([20], p. 189). A marketing mix analysis will need to be undertaken separately for each market segment.

The marketing mix has seven components (the 7 Ps): product, price, place (distribution), promotion/communication, people, processes, and physical environment. Product, price, place, processes, people, and physical environment shape the value proposition (the benefits that a trial has to offer) whereas promotion/communication determines how a value proposition is communicated. The 7 Ps provide a format in which systematic learning can occur, aiding trialists’ understanding of what is (or is not) happening and of what can be done to improve their trial’s appeal. We will describe each component and then discuss the marketing research methods that can be applied to gain insights about the 7 Ps. Table 2 illustrates how each P applied to txt2stop.

**Table 2:** Txt2stop examples for the 7 Ps

**The 7 Ps**	**Examples from txt2stop**
**Product**	**Core trial specific:** Direct and indirect benefits to quitting smoking.
	**Core general:** Feelings of pride and self-worth for contributing to research and the social good.
	**Tangible:** Txt2stop offered the only mobile phone-based smoking cessation support intervention available at the time; systematic individualised support for time-scarce smokers who value convenience and anonymity. The txt2stop and London School of Hygiene and Tropical Medicine name and logo were part of the branding, conveying values of scientific integrity.
	**Augmented:** Txt2stop offered a free phone line to answer queries.
**Price (costs)**	**Psychological and inconvenience:** Concerns about the trial being a ‘scam’ and the possibility of having to provide a saliva sample.
**Promotion/communication**	**Mass media:** Posters, newspapers and radio adverts, YouTube video, internet banner, and Facebook page. **Interpersonal channels:** GPs, pharmacists, and smoking cessation services.
**Place**	Wherever the participant took his/her phone.
**People**	Phone staff were selected on the basis of their communication skills, knowledge of trial procedures, and the ability to communicate empathy.
**Physical environment**	Not applicable – txt2stop provided its service via mobiles and recruited over the phone. Participants had on the most part no physical contact with the London School of Hygiene and Tropical Medicine, where the trial was based. A minority visited the school to provide a saliva sample.
**Process**	Txt2stop streamlined its processes by registering and randomising over the phone, keeping the duration of calls to a minimum and allowing consent by SMS.

### Product

The product is the trial itself, participants need to be “sold” trial participation and are interested in benefits and not mere attributes. A product (trial) can be conceived on three levels: core, tangible (actual), and augmented [21]. Understanding the three levels assists in the development of a marketing strategy.

The core product is the benefits participants are really buying into [22]. One could also think of trial participation in general as a core product, in as far as it can provide participants with a sense of pride and self-worth for contributing to a good cause – research. One can therefore distinguish between trial-specific and general core benefits. Appealing only to the general core benefits may not be enough to motivate trial participation. People are motivated by a desire to help others but this is often conditional on perceiving some personal benefit or at least no significant disadvantage to the self (conditional altruism) [25].

The tangible product is the actual product or service, including its features, quality, and brand. The augmented product is the add-on extras; tangible objects or services that support trial participation. These related benefits increase the attractiveness of trial participation, by improving the overall quality of the trial experience.

### Price (costs)

Price refers to more than financial cost; it is what participants must give up to partake in a trial. There are direct costs associated with joining a trial (entry costs), largely covered by the clinical trials literature under barriers to participation and can include psychological, physical, time, inconvenience, and monetary costs [2,25-27]. Not all perceived costs may be real, as they are based on participant misconceptions about clinical trials.

### Promotion and communication

Promotion covers the activities used to signal the merits of the other six components of the marketing mix. Effective signalling requires an appropriate message content, framing of messages, choice of channels, and mode of communication^a^[3,23,28,29]. Various media can be used, but each medium has its strengths and weaknesses. One must consider the costs, size, and type of audiences reached by each type of medium and determine the suitability of the vehicle for the message content and target audience [22].

Effective communication requires educating participants about the trial accrual process and addressing misconceptions. The availability and quality of information is an important factor influencing recruitment [2]. Patients have refused to join trials for not understanding randomisation or for fear of being a “guinea pig” [30].

### Place

Place is where and when the interaction with the trial’s product/service occurs and convenience is key [31]. From the trial’s perspective, place includes the distribution channel through which the trial’s product/service becomes available to the participant, which can involve the collaboration of interdependent organisations and middlemen [22], like hospitals or specialised centres. Participants’ preference for certain environments (for example, university hospitals over general hospitals), can affect trial participation [32].

### People

To varying degrees, trials have a people-led service component. Participants come in contact with staff such as clinicians, nurses, and assistants. The selection, training, and motivation of these “trial representatives” will impact upon recruitment. Through their empathy, competence, and courteous manner, the trial’s people shape expectations about the experience and the perceived quality of the offering. They play a central role in building trust and commitment between the trial and its participants, to such an extent that some trials have reported participants joining because they were impressed with the recruiter [33]. Participants who come to trust staff perceive lower risks to participation [34], and communication between the physician and the participant eases concerns about treatment “costs” and influences trial accrual [35-37].

### Physical environment

The physical environment within which a trial is delivered provides participants with cues and assurances about its trustworthiness and scientific integrity. Participants will have greater confidence in a trial conducted in a well-cared for facility.

### Process

Processes are the actions required of participants to participate in the trial. Here, ease of use is a key factor. For example, the nature of the consent process can be a reason for participants refusing to participate [2]. Trial protocols can be problematic if the regimen is difficult to follow, tedious [38], or deemed excessive [27].

### Marketing research methods: researching the marketing mix (7 Ps)

While marketing information is needed across the 7 Ps to understand why a trial might be underperforming, some clinical trials face specific challenges: budget limitations and the lack of marketing expertise impose constraints. Nevertheless, research need not be overcomplicated nor expensive and can be conducted in informal but effective ways [39]. Secondary data research, primary qualitative (causal observation, personal interviews, and focus groups), primary quantitative (i.e., questionnaires), and nested controlled trials to empirically test the validity of different marketing material or interventions are all possible research techniques [13,22,38,39].

### TASK 3 – performance evaluation

A trial must monitor and evaluate its performance to increase the probability that its objectives will be met. Recruitment should be tracked on an on-going basis and evaluated against goals [22], and the measures used should generate data on the cost effectiveness of marketing interventions. Findings must result in learning for the next campaign and provide input into future decisions [40]. Typical indicators for a trial would include: volume and cost per registration, consent, and randomisation, according to media and advert/intervention type.

### TASK 4 – implementing change

Having identified where problems lie and how these might be resolved, change initiatives can occur on two levels: at the level of single interventions and on a broader strategic level.

At the level of single intervention, change initiatives could include new or corrective measures to be undertaken within a trial’s marketing mix. These could improve promotion or communication by increasing the persuasiveness of trial messages, or stakeholders’ awareness and appreciation of the product benefits as well as their understanding of trial processes. In addition, it could be desirable to strive to improve participants’/collaborators’ trial experiences by addressing shortcomings in the place, people, physical environment, and process components of the marketing mix and by alleviating or compensating for the costs of trial participation/collaboration.

Broader strategic change could involve adopting a holistic view to improving recruitment by choosing the optimal mix of interventions and best strategic approach^b^, i.e., which interventions are worth introducing, modifying, or abandoning for the most productive use of resources. It is wise for such decisions to be made on the basis of marketing performance data, the expected cost effectiveness of the changes, as well as factors related to the trial’s resources, capabilities, and priorities.

### The txt2stop experience through the lens of the recruitment optimisation model

We have presented the recruitment optimisation model’s marketing concepts and processes, and now we will explain the txt2stop experience using the model’s framework. Our purpose is to illustrate how the model can be applied as a conceptual framework for guiding a flow of improvements to recruitment practices. We acknowledge that the model is being applied retrospectively.

### Recruitment phase monitoring

Eight months into recruitment (October 2007 to June 2008) and the trial was 250 participants short of target; 4,950 subjects were still to be recruited. Yet, 937 people had expressed an interest in the trial but had not yet registered (i.e., had completed phase 1 without further progression). A further 1,302 smokers had registered but had not given or had refused to give their consent, they had completed phase 2 with no further progression. Only 33% of eligible participants were consenting. Interventions were needed across all phases in order to get more people to express an interest and, crucially, to increase the probability that larger percentages of those expressing an interest would register and consent.

### Marketing research and findings

Below, we summarise the main lessons learnt from the marketing research under each of the 7 Ps and describe the changes that followed. We do this in a table format in the interest of brevity and clarity (Table 3).

**Table 3:** The 7 Ps lessons learned and changes made

**The 7 Ps**	**Lessons learned and changes made**
**Product**	EH reviewed the clinical trials and smoking cessation literature using the MEDLINE database and key search terms “recruitment and trials”, “smoking cessation”, and years 1960 to 2007. A range of reasons for participating in trials was identified from the literature.
	**General core benefits:** The opportunity to receive an intervention not otherwise available and the satisfaction of contributing to research [33,41]. **Trial specific core benefits:** The prospects of saving money, of improved health, of wanting to avoid damage to an unborn baby, and of quitting for one’s loved ones.
**Price (cost)**	Insights from social psychology theories were used to influence potential participants’ perceptions of the psychological costs to (non) participation [42].
	**Social validation theory:** Knowing that others similar to oneself have joined can provide reassurance that joining is the right decision [43].
	**Norms of reciprocity:** Receiving small financial and other incentives can increase participants’ willingness to cooperate [44].
	**Theory of scarcity:** Scarcity can act a heuristic for the perceived value of a medical intervention [45].
	£5 was sent to prospective participants with their covering letter to induce norms of reciprocity and to dismiss concerns that the trial could be a scam (i.e., charge for text messages received) [46]. Text messages were sent to participants who had registered and were eligible to join, to remind them that they could consent and that only 300 places were left (as was the case at the time) [47].
	***To smokers***
**Promotion communication**	Theories of persuasion suggest testimonials can generate positive responses [28,48]. Qualitative feedback from participants about their experience with txt2stop was obtained and with permission used in text messages sent to potential participants who had received the trial information but had not yet consented.
	The study information was personalised, shortened, and simplified, as the literature on trial participation suggested people might be more responsive to such types of communication [2,35,49]. The content was amended to mention the product tangible and general core benefits, that trial participation offered the possibility of trying a free and novel service, and that the research could be used by the NHS [50]. To signal trustworthiness all correspondence included university logos and was personally signed [3,29,36].
	New newspaper advertisements were prepared to increase public interest. These promoted the benefits to quitting (product core benefits) and used testimonials to maximise the impact of the message. Previous advertisements merely made people aware of txt2stop.
	***To health care providers***
	Smoking cessation literature suggested health care professionals can act as important triggers for quit attempts [51]. Yet, very few participants had been recruited through health care providers. GPs interviewed reported that remembering and workload were issues, so recruitment through GP surgeries was redesigned. GPs were reimbursed via the PCRN for admin staff writing to smokers on their lists inviting them to take part in the trial. Pharmacists reported wanting paying for recruitment but at the time there was no mechanism for funding this. Smoking cessation services received funds for smokers they helped to quit and so they had no incentive to refer smokers on. No further effort was put into promotions through these channels. However, a txt2stop link was placed on the NHS smoking cessation websites.
**Place**	Not applicable to txt2stop, interaction occurred over the phone.
**People**	Adequately managing “moments of truth” in the participant/staff interaction, such as responding convincingly to a challenging query, and minimising “response rate tyranny” (annoyance and harassment) is key to engaging potential participants [9,52].
	Through informal observations and through semi-structured phone interviews held with a sample of individuals who had refused consent, participants’ concerns and misconceptions that were unique to txt2stop were spotted. Staff received training to address concerns such as that DNA testing was secretly linked to the provision of a saliva sample. Staff also received training on how to deal with awkward and busy participants and on how to show empathy towards those who were allocated to the control group while reminding them they still had an important role to play in the trial. Capitalising on the good rapport that phone staff had with participants, staff asked participants to promote the trial by word of mouth.
**Physical environment**	Not applicable to txt2stop, interaction occurred over the phone.
**Process**	Online registration (instead of just by phone) was made available for busy and hard to reach individuals and promoted in the voice messages left on their mobiles and also by a series of text messages.

### Performance evaluation

On registration, participants were asked where they had heard of txt2stop. A record was kept of the promotional expenditure pertaining to each communication campaign. Recruitment was monitored across sources (radio, newspaper, GP surgeries, pharmacies, smoking cessation clinic), within sources (e.g., different radio stations, newspapers, or GP surgeries), and according to advert type, where applicable. Discrepancies were noted in performance in terms of volume and cost per randomisation. This resulted in strategy level modifications, as funds were pulled from underperforming media and campaigns, and reallocated. Poorly performing media, such as the Internet banner and social media, were immediately dropped. Instead, the very high volume of enquiries generated by radio, coupled with its relative cost effectiveness and quick turnaround, made radio advertising the priority medium, and the intensity and frequency of radio campaigns was increased. The most cost effective radio stations and advert type were identified and reemployed as necessary. While, on average, the newspaper adverts were more cost effective than the radio (largely due to the free adverts in some newspapers), the comparatively low volume of randomisations generated, coupled with the unpredictable variability in cost per randomisation according to newspaper type meant only the most productive and the free adverts were kept running.

### Recruitment results

**Figure 4:**
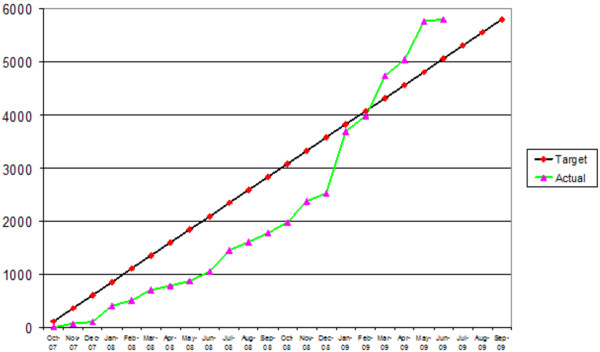
Trial recruitment target vs. actual.

**Figure 5:**
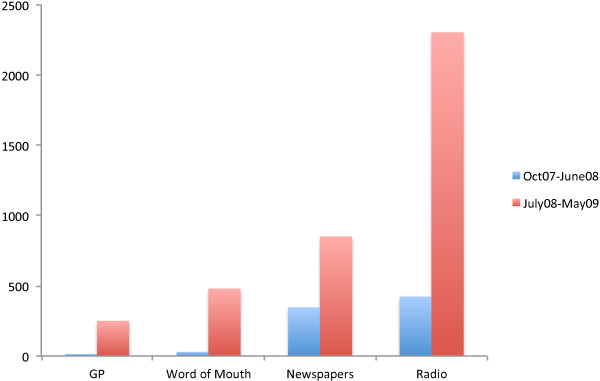
Number of participants recruited according to source.

Combined, the changes resulted in substantial improvements in recruitment. Most of the discussed intervention level changes (across the 7 Ps) were implemented in May/June 2008. Between July and September 2008, radio and newspaper advertisements ceased and the relatively modest but almost immediate increase in recruitment of 510 participants that can be observed (Figure 4) is primarily attributable to recruitment via word of mouth and to interventions introduced to increase consent and registration rates among participants who had already expressed an interest but had not yet registered and/or consented (stuck in step 1 or 2 of phase 2); results from nested control trials that we ran support this. Testimonial text messages sent to participants who had registered but not consented proved effective as did the scarcity messages [47] and sending £5 with the study consent letter [46]. Text messages sent to potential participants, reminding them of the option of online registration, increased registrations [46]. Following the completion of the nested trials, the tested interventions became standard recruitment procedures. Subsequently, a revised radio and newspaper promotional campaign was developed for January 2009, drawing on the experience gained in identifying effective adverts and media. This was run in the New Year to coincide with smokers making new year’s resolutions to quit and coupled with the effect of the other downstream changes already discussed, the campaign resulted in the dramatic increase in recruitment that can be observed from January 2009 until August 2009. Over the final 10 months of recruitment, recruitment levels more than quadrupled, the average cost per person randomised fell to a 1/3 over the last two quarters, and the proportion of eligible participants joining the trial rose from 33% to 57%, exceeding the 50% target. The trial finished by recruiting four months ahead of schedule.Figure 5 shows the number of participants recruited according to source, before and after the implemented changes. Meaningful improvements in the recruitment performance of newspaper adverts, GPs, and in the “word of mouth” media can be noted. The latter is testament to participants having a positive experience of the trial. However, recruitment was largely through radio adverts.

## Discussion

### Summary of the recruitment optimisation model

The model is circular and thus learning can begin at any stage. At its heart lie three interdependent processes: recruitment phase monitoring, marketing research, and performance evaluation. Recruitment phase tracking locates bottlenecks in the recruitment process while marketing research provides insights into the nature of the problems and how these can be overcome. Performance evaluation allows trialists to determine the efficacy and cost effectiveness of a trial’s marketing interventions and strategies and to allocate resources accordingly. These three processes provide the knowledge for making improvements to existing interventions/strategies and for introducing new bespoke solutions. The changes will shape the target market’s perception and awareness of the trial, their evaluation of costs and benefits, and the extent to which their needs are satisfied. The effects will be reflected in recruitment outcome measures (volume of randomisations, cost per randomisation, registration and consent rates) generating new sets of (monthly) data, which can feed back into the system, forming the basis of a new learning cycle (Figure 3).

### Comparison with existing literature

The clinical trials literature has struggled to identify the factors and practices responsible for successful recruitment. Quantitative approaches that have used broad data sets have encountered difficulties in reaching synthesis, with comparative analysis producing little insight due to the complexity and marked variability of the trials under consideration [1,5,53]. Qualitative studies have provided more detailed descriptive accounts but given the context specificity of many trials, the scope for a direct transfer of lessons learned to other trials can be limited [17,54,55].

Both approaches have been hampered by the lack of a conceptual framework to assist with sense making. There is a recognised and urgent need for models that provide relevant material for those undertaking clinical trials [7].

**Figure 6:**
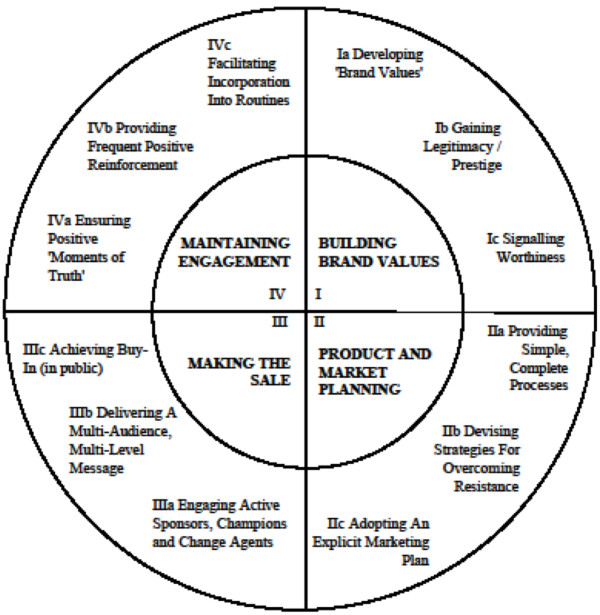
**The Francis et al. reference model**[9].

Francis et al. [9] (Figure 6) proposed a reference model for marketing a trial which has served as a tentative diagnostic tool [56]. We argue the model is most valuable as a reference tool rather than as a dynamic diagnostic tool. While the Francis model suggests domains in which trial management needs to form marketing strategies, it does not pinpoint the core practices needed to support learning. What is called for is a model that recognises the task of improving recruitment as an emergent process where any deviations from expectations or plans can be spotted and analysed, and remedial action can be undertaken. The recruitment optimisation model aims to fill this gap. The importance of this is illustrated by the txt2stop trial, which fully recruited to its pilot trial within two weeks; thus, the poor initial recruitment to the main trial was unexpected.

### Study limitations and subsequent areas for future research

Although firmly grounded in marketing theory and the clinical trials literature, the recruitment optimisation model is founded on a post hoc analysis of a single case study (txt2stop) and it was not feasible to quantify the precise extent to which the learning processes enacted were responsible for all the improvements in recruitment described. The model is best viewed as a tentative framework to be tested and validated by other types of trials.

The literature searches completed by EH were done within the budget and time constraints of a trial seeking solutions to trial recruitment and did not meet the standards of a systematic review of evidence. Only one database (Medline) was searched and a more exhaustive search strategy would be likely to have resulted in the identification of further relevant studies. However, systematic reviews of the relevant trial participation literature have since been completed [26,57].

## Conclusions

Improving recruitment performance requires a learning process. The recruitment optimisation model suggests the key practices (marketing research, monitoring and evaluating performance) and marketing constructs (the 7 Ps) that are necessary to guide the learning experience. The 7 Ps provide a format within which learning can occur. Therefore, the main contributions of this paper are to advance the clinical trial literature’s understanding of i) what trialists need to learn about, ii) what helps them learn better, and iii) how learning can translate into improved action and increased recruitment. There are potentially significant implications for clinical trials and funding bodies. The model’s application could optimise or help improve the recruitment of clinical studies resulting in more efficient trials.

## End notes

^a^For mode we refer to the distinction that can be drawn between the two different ways of gaining knowledge, the paradigmatic and the narrative, and in particular we draw attention to the merits of narrative communication. See Hinyard & Kreuter, [28].

^b^For strategy we refer to broad decisions relating to a trial’s recruitment process, for instance whether to recruit using mass media tools for promotion or in-person approaches. Strategies themselves consist of individual interventions, which determine how a strategy is executed. Interventions can include anything from the use of posters to participant letters, radio adverts, or the use of intermediaries (GPs).

## Competing interests

The author(s) declare that they have no competing interests.

## Authors’ contributions

LG wrote the paper with CF and DF. LG conducted the interview with the trial investigator (CF), reviewed the marketing literature, and conceived the recruitment optimisation model with input from DF and CF. CF, RK, SR, and the whole txt2stop team generated all the ideas to improve recruitment and implemented all the changes to recruitment procedures described in the paper. SR monitored the data on trial recruitment, the effectiveness of different promotions, and recruitment phase monitoring. EH conducted literature searches and summarised the literature on factors influencing quitting and factors influencing trlal participation. OO conducted interviews with pharmacists. DF introduced CF to marketing principles and his adaptation of these to clinical trials, which helped guide some of the changes to recruitment procedures described. All authors read and approved the final manuscript.

## References

[B1] McDonald AM Knight RC Campbell MK Entwistle VA Grant AM Cook JA Elbourne DR Francis D Garcia J Roberts I Snowdon C What influences recruitment to randomised controlled trials? A review of trials funded by two UK funding agencies Trials 2006 7 9 10.1186/1745-6215-7-9 16603070 PMC1475627

[B2] Ross S Grant A Counsell C Gillespie W Russell I Prescott R Barriers to participation in randomised controlled trials: a systematic review J Clin Epidemiol 1999 52 12 1143 1156 10.1016/S0895-4356(99)00141-9 10580777

[B3] Patel M Doku V Tennakoon L Challenges in recruitment of research participants Adv Psychiatr Treat 2003 9 229 238 10.1192/apt.9.3.229

[B4] Sully BG Julious SA Nicholl J A reinvestigation of recruitment to randomised, controlled, multicenter trials: a review of trials funded by two UK funding agencies Trials 2013 14 1 166 10.1186/1745-6215-14-166 23758961 PMC3691846

[B5] Mapstone J Elbourne D Roberts I Strategies to improve recruitment to research studies Cochrane Database Syst Rev 2007 2 MR000013 17443634 10.1002/14651858.MR000013.pub3

[B6] Medical Research Council Clinical Trials for Tomorrow 2003 London: MRC

[B7] Farrell B Kenyon S Shakur H Managing clinical trials Trials 2010 11 1 78 10.1186/1745-6215-11-78 20626885 PMC2917433

[B8] Heiney P Arp Adams S Drake BF Bryant LH Bridges L Hebert JR Successful subject recruitment for a prostate cancer behavioral intervention trial Clinical Trials 2010 7 4 411 417 10.1177/1740774510373491 20571136 PMC2959175

[B9] Francis D Roberts I Elbourne DR Shakur H Knight RC Garcia J Snowdon C Entwistle VA McDonald AM Grant AM Campbell MK Marketing and clinical trials: a case study Trials 2007 8 37 10.1186/1745-6215-8-37 18028537 PMC2212650

[B10] Donovan J Mills N Smith M Brindle L Jacoby A Peters T Frankel S Neal D Hamdy F Quality improvement report: Improving design and conduct of randomised trials by embedding them in qualitative research: ProtecT (prostate testing for cancer and treatment) study. Commentary: presenting unbiased information to patients can be difficult BMJ 2002 325 7367 766 770 10.1136/bmj.325.7367.766 12364308 PMC1124277

[B11] Smith A Social marketing: an evolving definition Am J Health Behav 2000 24 1 11 17 10.5993/AJHB.24.1.3

[B12] Hastings G Haywood A Social marketing and communication in health promotion Health Promot Int 1991 6 2 135 145 10.1093/heapro/6.2.135

[B13] Grier S Bryant CA Social marketing in public health Annual Review of Public Health 2005 26 319 339 10.1146/annurev.publhealth.26.021304.144610 15760292

[B14] Boyle RG Enstad C Asche SE Thoele MJ Sherwood NE Evaluating strategies and costs to recruit smokeless tobacco users Addict Behav 2007 32 12 3088 3092 10.1016/j.addbeh.2007.06.005 17602843

[B15] Dyas JV Apekey T Tilling M Siriwardena AN Strategies for improving patient recruitment to focus groups in primary care: a case study reflective paper using an analytical framework BMC Med Res Methodol 2009 9 65 10.1186/1471-2288-9-65 19772603 PMC2759948

[B16] Gabbay M Thomas J When free condoms and spermicide are not enough: barriers and solutions to participant recruitment to community-based trials Control Clin Trials 2004 25 4 388 399 10.1016/j.cct.2004.06.004 15296813

[B17] Atherton H Banks D Harbit R Long L Chadd F Hay P Kerry S Simms I Oakeshott P Recruitment of young women to a trial of chlamydia screening – as easy as it sounds? Trials 2007 8 41 10.1186/1745-6215-8-41 18053199 PMC2212649

[B18] Free C Whittaker R Knight R Abramsky T Rodgers A Roberts IG Txt2stop: a pilot randomised controlled trial of mobile phone-based smoking cessation support Tob Control 2009 18 2 88 91 10.1136/tc.2008.026146 19318534

[B19] Kolb DA Experiential Learning: Experience as the Source of Learning and Development 1984 Englewood Cliffs, NJ: Prentice Hall

[B20] Black DR Blue CL Coster DC Chrysler LM Corporate social marketing: message design to recruit program participants Am J Health Behav 2002 26 3 188 199 10.5993/AJHB.26.3.4 12018755

[B21] Kotler P Armstrong G Principles of Marketing 2010 13 US: Pearson Education 613

[B22] Kotler P Roberto N Lee N Social Marketing: Improving the Quality of Life 2002 Thousand Oaks, CA: SAGE

[B23] Formoso G Marata AM Magrini N Social marketing: should it be used to promote evidence-based health information? Soc Sci Med 2007 64 4 949 953 10.1016/j.socscimed.2006.09.022 17141384

[B24] Edwards P Arango M Balica L Cottingham R El-Sayed H Farrell B Fernandes J Gogichaisvili T Golden N Hartzenberg B Husain M Ulloa MI Jerbi Z Khamis H Komolafe E Laloë V Lomas G Ludwig S Mazairac G Muñoz Sanchéz Mde L Nasi L Olldashi F Plunkett P Roberts I Sandercock P Shakur H Soler C Stocker R Svoboda P Trenkler S CRASH trial collaborators: Final results of MRC CRASH, a randomised placebo-controlled trial of intravenous corticosteroid in adults with head injury? Outcomes at 6 months Lancet 2005 365 9475 1957 1959 15936423 10.1016/S0140-6736(05)66552-X

[B25] McCann SK Campbell MK Entwistle VA Reasons for participating in randomised controlled trials: conditional altruism and considerations for self Trials 2010 11 1 31 10.1186/1745-6215-11-31 20307273 PMC2848220

[B26] McCann S Campbell M Entwistle V Recruitment to clinical trials: a meta-ethnographic synthesis of studies of reasons for participation J Health Serv Res Policy 2013 18 4 233 241 10.1177/1355819613483126 23986530

[B27] Grunfeld E Zitzelsberger L Coristine M Aspelund F Barriers and facilitators to enrollment in cancer clinical trials Cancer 2002 95 7 1577 1583 10.1002/cncr.10862 12237928

[B28] Hinyard LJ Kreuter MW Using narrative communication as a tool for health behavior change: a conceptual, theoretical, and empirical overview Health Educ Behav 2007 34 5 777 792 10.1177/1090198106291963 17200094

[B29] Edwards P Roberts I Clarke M DiGuiseppi C Pratap S Wentz R Kwan I Increasing response rates to postal questionnaires: systematic review BMJ 2002 324 7347 1183 10.1136/bmj.324.7347.1183 12016181 PMC111107

[B30] Trauth JM Musa D Siminoff L Jewell IK Ricci E Public attitudes regarding willingness to participate in medical research studies J Health Soc Policy 2000 12 2 23 43 10.1300/J045v12n02_02 11184441

[B31] Newington L Metcalfe A Factors influencing recruitment to research: qualitative study of the experiences and perceptions of research teams BMC Med Res Methodol 2014 14 1 10 10.1186/1471-2288-14-10 24456229 PMC3903025

[B32] Verheggen FWSM Nieman F Jonkers R Determinants of patient participation in clinical studies requiring informed consent: why patients enter a clinical trial Patient Educ Couns 1998 35 2 111 125 10.1016/S0738-3991(98)00060-3 10026554

[B33] Chang BH Hendricks AM Slawsky MT Locastro JS Patient recruitment to a randomized clinical trial of behavioral therapy for chronic heart failure BMC Med Res Methodol 2004 4 8 10.1186/1471-2288-4-8 15090073 PMC404462

[B34] Lowton K Trials and tribulations: Understanding motivations for clinical research participation amongst adults with cystic fibrosis Soc Sci Med 2005 61 8 1854 1865 10.1016/j.socscimed.2005.03.039 15913858

[B35] Albrecht TL Eggly SS Gleason ME Harper FW Foster TS Peterson AM Orom H Penner LA Ruckdeschel JC Influence of clinical communication on patients’ decision making on participation in clinical trials J Clin Oncol 2008 26 16 2666 2673 10.1200/JCO.2007.14.8114 18509178 PMC3807688

[B36] Dixon-Woods M Tarrant C Why do people cooperate with medical research? Findings from three studies Soc Sci Med 2009 68 12 2215 2222 10.1016/j.socscimed.2009.03.034 19394741

[B37] Fuertes JN Mislowack A Bennett J Paul L Gilbert TC Fontan G Boylan LS The physician-patient working alliance Patient Educ Couns 2007 66 1 29 36 10.1016/j.pec.2006.09.013 17188453

[B38] Prescott RJ Counsell CE Gillespie WJ Grant AM Russell IT Kiauka S Colthart IR Ross S Shepherd SM Russell D Factors that limit the quality, number and progress of randomised controlled trials Health Technol Assess 1999 3 20 1 143 10683591

[B39] Andreasen A Marketing Research that Wont’t Break the Bank: A Practical Guide to Getting the Information You Need 2002 2 San Francisco: Jossey-Bass

[B40] Fill C Essentials of Marketing Communications 2011 Englewood Cliffs, NJ: Financial Times Prentice Hall

[B41] Jenkins V Fallowfield L Reasons for accepting or declining to participate in randomized clinical trials for cancer therapy Br J Cancer 2000 82 11 1783 1788 10.1054/bjoc.2000.1142 10839291 PMC2363224

[B42] Groves RM Cialdini RB Couper MP Understanding the decision to participate in a survey Public Opin Q 1992 56 4 475 495 10.1086/269338

[B43] Lovato LC Hill K Hertert S Hunninghake DB Probstfield JL Recruitment for controlled clinical trials: literature summary and annotated bibliography Control Clin Trials 1997 18 4 328 352 10.1016/S0197-2456(96)00236-X 9257072

[B44] Martinson BC Lazovich D Lando HA Perry CL McGovern PG Boyle RG Effectiveness of monetary incentives for recruiting adolescents to an intervention trial to reduce smoking Prev Med 2000 31 6 706 713 10.1006/pmed.2000.0762 11133338

[B45] Ditto PH Jemmott JB From rarity to evaluative extremity: effects of prevalence information on evaluations of positive and negative characteristics J Pers Soc Psychol 1989 57 1 16 2754601 10.1037//0022-3514.57.1.16

[B46] Free C Hoile E Robertson S Knight R Three controlled trials of interventions to increase recruitment to a randomized controlled trial of mobile phone based smoking cessation support Clinical Trials 2010 7 3 265 273 10.1177/1740774510367687 20484492

[B47] Free CJ Hoile E Knight R Robertson S Devries KM Do messages of scarcity increase trial recruitment? Contemp Clin Trials 2011 32 1 36 39 10.1016/j.cct.2010.09.002 20840874

[B48] Slater M Dillard J, Pfau M Involvement as goal-directed strategic processing: Extending the elaboration likelihood model The Persuasion Handbook: Developments in Theory and Practice 2002 Thousand Oaks, CA: Sage

[B49] Watson JM Torgerson DJ Increasing recruitment to randomised trials: a review of randomised controlled trials BMC Med Res Methodol 2006 6 1 34 10.1186/1471-2288-6-34 16854229 PMC1559709

[B50] Williams B Entwistle V Haddow G Wells M Promoting research participation: why not advertise altruism? Soc Sci Med 2008 66 7 1451 1456 10.1016/j.socscimed.2007.12.013 18222579

[B51] Murray RL Lewis SA Coleman T Britton J McNeill A Unplanned attempts to quit smoking: missed opportunities for health promotion? Addiction 2009 104 11 1901 1909 10.1111/j.1360-0443.2009.02647.x 19681806

[B52] Kolar T Kolar I What respondents really expect from researchers Eval Rev 2008 32 4 363 391 10.1177/0193841X07306953 18591708

[B53] Sarre G PROSPeR. An Analytical Framework for Planning and Sustaining Recruitment to Research Studies in Primary Care Based on Evidence from the Literature 2008 http://webarchive.nationalarchives.gov.uk/20100218141456/nspcr.ac.uk/publications/prosper2.pdf

[B54] Ziebland S Featherstone K Snowdon C Barker K Frost H Fairbank J Does it matter if clinicians recruiting for a trial don’t understand what the trial is really about? Qualitative study of surgeons’ experiences of participation in a pragmatic multi-centre RCT Trials 2007 8 4 647 660 10.1186/1745-6215-8-4 PMC1794540 17257440

[B55] Toledo L McLellan-Lemal E Arreola S Campbell C Sutton M African-American and Hispanic perceptions of HIV vaccine clinical research: a qualitative study Am J Health Promot 2014 In press 10.4278/ajhp.130125-QUAL-48 24432823

[B56] Campbell MK Snowdon C Francis D Elbourne D McDonald AM Knight R Entwistle V Garcia J Roberts I Grant A Grant A STEPS group Recruitment to randomised trials: strategies for trial enrolment and participation study. The STEPS study Health Technology Assessment 2007 11 48 iii, ix-105 17999843 10.3310/hta11480

[B57] Treweek S Lockhart P Pitkethly M Cook JA Kjeldstrøm M Johansen M Taskila TK Sullivan FM Wilson S Jackson C Jones R Mitchell ED Methods to improve recruitment to randomised controlled trials: Cochrane systematic review and meta-analysis BMJ Open 2013 3 2 pii:e002360 10.1136/bmjopen-2012-002360 PMC3586125 23396504

